# Defining the Data set Defines the QSAR Claim

**DOI:** 10.1021/acs.jcim.6c00514

**Published:** 2026-02-27

**Authors:** Manal A. Nael, Laxman M. Alakonda, Khaled M. Elokely

**Affiliations:** † Department of Pharmaceutical Chemistry, Faculty of Pharmacy, Tanta University, Tanta 31527, Egypt; ‡ Division of Pharmaceutical Sciences, School of Pharmacy, 4416 University of Wyoming, 1000 E. University Avenue, Laramie, Wyoming 82071, United States

## Abstract

Machine learning has greatly expanded QSAR modeling, but predictive claims still depend on choices that are rarely documented: how chemicals are represented, how end points are defined, and how evaluations are designed. In the era of benchmarks and foundation models, inconsistent standardization, unclear rules for combining measurements, and hidden information leakage routinely inflate reported performance while obscuring weaknesses that matter for real-world applications. We propose data set contracts: executable, auditable documents that explicitly declare chemical processing rules, end point definitions, aggregation logic, data splits, and leakage diagnostics for the intended prediction scenario. These contracts are feasible with current open-source tools and would shift the field from architecture-centric comparisons toward claims that are transparent, reproducible, and trustworthy.

## Graphical abstract

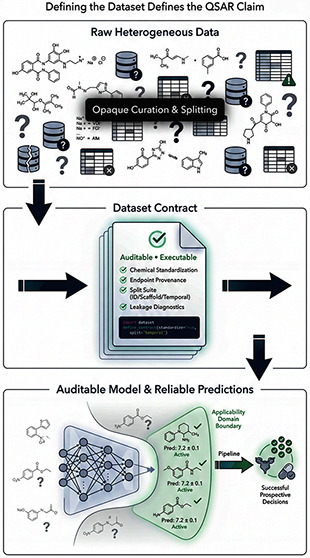

QSAR has always been a paradoxical discipline: it promises generalizable predictions from molecular structure, yet its validity depends entirely on the quality and meaning of the experimental data behind those predictions. The Hansch–Fujita formulation made this explicit by anchoring models in comparable chemical series and well-defined end points rather than algorithmic novelty alone.^1^ Over time, the field developed a culture of validation discipline, formalized for regulatory audiences by the OECD principles requiring a defined end point, an unambiguous algorithm, an applicability domain, appropriate measures of goodness-of-fit and robustness, and, where possible, mechanistic interpretation.^2,3^ The Tropsha–Golbraikh tradition likewise warned that optimistic internal metrics can arise from redundancy, selection bias, and weak external validation rather than genuine predictive power.^4–6^

Yet the past decade has shifted community attention from this foundational work toward benchmark performance and architectural innovation.^7–10^ Public resources such as ChEMBL enabled large-scale data set assembly, but they also aggregate assays with different designs, conditions, and reporting standards. This complexity is easy to overlook once data appear as clean spreadsheets rather than the messy experimental records they summarize.^11,12^ Modeling pipelines evolved in parallel: from handcrafted descriptors to fingerprint-based machine learning, then to deep learning on graphs and strings, and most recently to foundation models built on pretrained molecular encoders.^13–16^ Benchmark suites such as MoleculeNet standardized evaluation and accelerated method comparison, but they also encouraged workflows in which data set preparation is treated as a solved problem while architecture and training strategy take center stage.^17^

This shift raises a pointed question: has machine learning made rigorous curation obsolete? The answer is no. Scale and representation learning can absorb some noise, but they cannot resolve ambiguity in the underlying measurements. Worse, high-capacity models can fit subtle artifacts (hidden patterns baked into how the data set was assembled) and still appear strong under convenient evaluation setups.

Four failure modes illustrate the problem.

## Chemical Identity: The Modeling Assumption Nobody Documents

A molecule is not a unique object in public data sets. Salt forms, mixtures, tautomers, charge states, aromaticity conventions, and stereochemical representations vary across sources and toolkits, altering fingerprints, graph topology, and nearest-neighbor relationships.^18^ Many studies compress these decisions into a single line (e.g., “standardized with RDKit”), even though modest rule changes can materially affect deduplication, split difficulty, and the effective learning problem. In some cases, standardization claims are supported by citations to papers that do not describe any standardization protocol, making the procedure not merely undocumented but unverifiable. When papers do not state whether models use submitted, standardized, or parent structures, and how stereochemistry and tautomers are handled, readers cannot determine what “duplicate”, “novel”, or “out-of-domain” means in practice.

## End-Point Definition Collapses under Label Aggregation

Public bioactivity data routinely combine measurements across assay formats, constructs, laboratories, and reporting conventions. Without explicit selection and transformation logic, a single modeled end point can conflate nonequivalent phenomena.^12^ Modern evaluation guidance emphasizes that mixing heterogeneous study designs and metrics undermines both comparability and relevance assessment.^19^ In practice, AI-era reporting often makes it difficult to reconstruct whether a model predicts an assay-specific readout, a target-level aggregation, or a multiprotocol mixture. A particularly insidious variant arises when binding affinity measures (e.g., K*_i_*, IC_50_) are pooled with functional readouts (e.g., EC_50_) under a single label, even though the former cannot distinguish mechanistic categories such as agonists from antagonists. Similarly, collapsing pharmacologically distinct categories (such as labeling partial agonists as full agonists) corrupts the decision boundary a classifier is meant to learn, yet is often dismissed as a pragmatic simplification.

## Overoptimistic Evaluation from Information Leakage

Leakage is now recognized across scientific machine learning as a major driver of irreproducible claims: models exploit unintended shortcuts when training and test sets are not meaningfully independent for the type of prediction being claimed.^20^ Chemistry data sets are clustered by scaffold series, vendor catalogs, and medicinal chemistry campaigns, so random splits often evaluate interpolation within analog neighborhoods rather than true generalization. Benchmark practice can reward memorization when train-test dependencies remain strong.^21^ Even scaffold splits can overestimate virtual screening performance when structurally distinct scaffolds remain highly similar in the representation space used for modeling.^22,23^ When prospective use is implied, time-based or temporal-analogue splits remain the defensible gold standard,^24^ yet many papers still rely on a single convenient split without quantifying residual similarity or describing likely leakage pathways.

## Average Metrics Hide the Cases That Matter Most

Activity cliffs (small structural changes producing large activity differences) expose where models fail locally even when global performance appears strong. Benchmarking studies show that many machine learning approaches struggle specifically on cliff compounds, revealing limitations masked by aggregate scores.^25^ Cliffs are also a curation problem: they can only be studied rigorously when chemical identity, duplicates, and label integrity are handled with sufficient care (Table 1).

**Table 1:** Common Failure Modes in AI-Era QSAR Modeling and How Dataset Contracts Address Them

failure mode	manifestation in current practice	consequence for claims and trustworthiness	addressed by data set contract through
Chemical identity: the undocumented modeling assumption	Vague or undocumented standardization (e.g., “standardized with RDKit”); variable handling of salts, tautomers, stereochemistry, and parent structures; citations to irrelevant papers used as proxies for missing protocols.	Readers cannot interpret what “duplicate”, “novel”, or “out-of-domain” actually means; splits and nearest neighbors become unreliable.	Explicit identity mappings + specification of which representation is used for modeling, deduplication, and splitting.
End-point definition collapses under label aggregation	Unspecified mixing of heterogeneous assays, formats, laboratories, and reporting conventions into a single modeled label; conflation of binding affinity and functional end points; collapsing of pharmacologically distinct categories (e.g., partial agonists labeled as full agonists).	Model predicts a semantically ambiguous mixture rather than a well-defined biological phenomenon.	Structured end point provenance documenting selection, transformation, aggregation logic, and source identifiers.
Overoptimistic evaluation from information leakage	Random or scaffold splits on clustered data; unquantified train-test similarity; hidden dependencies from shared series or campaigns.	High benchmark scores often reflect interpolation or memorization rather than the prospective generalization claimed.	Labeled split suite + quantitative leakage diagnostics (similarity metrics + leakage pathway narrative).
Average metrics hide the cases that matter most	Reliance on global statistics while ignoring activity cliffs and local failure modes.	Models appear strong overall but fail on the structurally similar compounds most critical for lead optimization and safety.	Transparent chemical identity handling + end-point integrity, enabling rigorous cliff analysis and applicability domain assessment.

These failure modes share a common root: in AI-era QSAR, the data set and evaluation procedure act as a hidden set of rules that defines what the model actually learns, yet these rules are rarely published with the same rigor as the model itself. The remedy is straightforward: the field should adopt data set contracts (explicit, executable artifacts that make curation and evaluation decisions auditable).

A data-set contract is the minimal set of artifacts required for a performance claim to be interpretable, reproducible, and comparable. It is not a mandate for a universal standardization policy or a single splitting method; it is a requirement that the choices made are declared, versioned, and executable, because those choices define what the model is learning and what a reported metric can legitimately support (Table 2).

**Table 2:** Core Components of a Dataset Contract for QSAR and Molecular Machine Learning Studies

component	description	primary purpose
executable manifest	Code or script that regenerates the exact processed data set and split folds from raw inputs using declared rules and tool versions.	Full reproducibility of the learning task.
chemical identity mappings	Explicit mappings (submitted to standardized to parent) with rules for salts, mixtures, tautomers, stereochemistry, etc., and specification of which representation is used for each step.	Eliminates ambiguity in what constitutes a unique molecule or novel structure.
end-point provenance	Structured documentation of assay selection, aggregation logic, units, censoring, replicate handling, and source identifiers.	Ensures the modeled label has clear semantic meaning.
split suite	Multiple reproducible splits labeled by intent (in-distribution, scaffold/cluster, temporal) with published assignments or code.	Enables evaluation across relevant generalization regimes.
leakage diagnostics	Quantitative train-test similarity metrics + narrative of likely leakage pathways under the chosen representation.	Reveals whether performance reflects true extrapolation.
decision-context alignment	Explicit linkage of primary metrics to the intended real-world use case (i.e., matching the evaluation metric to how the model will be used in practice).	Grounds claims in practical decision-making relevance.
manual curation log	When curation involves manual decisions (such as literature-based verification of pharmacological labels), a structured annotation log with compound-level provenance rather than a narrative description alone.	Makes subjective curation steps auditable and reproducible.

These expectations are feasible today. Open, auditable curation pipelines exist for chemical structure processing and standardization at scale,^26,27^ and tools for constructing and auditing splits under leakage constraints are openly available.^28,29^ Journals need not endorse a single ruleset; they need only require that the ruleset used be declared in executable form.

For maximum impact with minimal additional burden, editors and reviewers can enforce a concise requirement: no QSAR paper should be accepted if the data set contract remains implicit. At minimum, manuscripts should include (i) a manifest that regenerates the data set from raw inputs, (ii) identity mappings and tool versions, (iii) end point provenance, (iv) published split assignments with intent labels, (v) leakage diagnostics, and (vi) structured logs for any manual curation steps. This expectation parallels existing norms for code and model deposition and would immediately raise the floor on comparability and trustworthiness. It also makes applicability domain operational: when identity, end point semantics, and the intended prediction scenario are explicit, domain statements become checkable rather than aspirational.^2,3^

Without data set contracts, reviewers must reverse-engineer curation decisions from ambiguous methods sections, a time-consuming process that, when it reveals fundamental flaws, often results in repeated revision cycles that burden authors, reviewers, and editors alike. Explicit contracts would surface these issues before submission, not after.

The AI era can either amplify QSAR’s historical strengths (clear end point semantics, explicit applicability constraints, and credible validation) or amplify its weaknesses by making hidden data set decisions harder to see and easier to exploit. Data set contracts offer the fastest, most practical path to ensuring the former. By making curation and evaluation intent explicit, the community can move from architecture-centric benchmark competitions toward claims that are interpretable, comparable, and genuinely useful for prospective decision-making. The tools exist. The question is whether the field will adopt them.
